# Quantitative Proteome Reveals Variation in the Condition Factor of Sea Urchin *Strongylocentrotus nudus* during the Fishing Season Using an iTRAQ-based Approach

**DOI:** 10.3390/md17070397

**Published:** 2019-07-05

**Authors:** Wen-Hui Shang, Jia-Run Han, Jia-Nan Yan, Yi-Nan Du, Yun-Sheng Xu, Chang-Feng Xue, Tie-Tao Zhang, Hai-Tao Wu, Bei-Wei Zhu

**Affiliations:** 1 School of Food Science and Technology, Dalian Polytechnic University, Dalian 116034, China; 2 College of Food Science and Engineering, Hainan Tropical Ocean University, Hainan 572022, China; 3 National Engineering Research Center of Seafood, Dalian 116034, China

**Keywords:** *Strongylocentrotus nudus*, seasonal variation, gonad index, condition factor

## Abstract

To investigate the variation in the condition factor of the sea urchin *Strongylocentrotus nudus* (*S. nudus*), gonads were collected in May (MAY), June (JUN), and July (JUL), at the beginning (AUG-b) and end of August (AUG-e). Sodium dodecyl sulfate polyacrylamide gel electrophoresis (SDS-PAGE) detection of the gonads revealed an obvious enhancement of the band at about 37 kDa from July, which was identified as transforming growth factor-beta-induced protein ig-h3 (TGFBI) by nanoLC-ESI-MS/MS. Gonadal proteins were identified by isobaric tagging for relative and absolute quantitation (iTRAQ), and regulation of the identified proteins in pairs of the collected groups was observed. A total of 174 differentially expressed proteins (DEPs) were identified. Seven of the DEPs showed significant correlations with both the gonad index (GI) and protein content. These correlations included 6-phosphogluconate dehydrogenase, decarboxylating isoform X2 (6PGD), CAD protein, myoferlin isoform X8, ribosomal protein L36 (RL36), isocitrate dehydrogenase [NADP], mitochondrial isoform X2 (IDH), multifunctional protein ADE2 isoform X3, sperm-activating peptides (SAPs) and aldehyde dehydrogenase, and mitochondrial (ALDH). However, TGFBI had no correlation with gonad index (GI) or protein content. 6PGD, IDH, multifunctional protein ADE2 isoform X3, and ALDH were shown to interact with each other and might play key roles in changing the condition factor of *S. nudus* gonads.

## 1. Introduction

The sea urchin, an aquatic animal belonging to the class Echinoidea, is distributed worldwide. *Strongylocentrotus nudus* (*S. nudus*), a member of the family Strongylocentrotidae, is an economically important species in northwestern Pacific countries, such as China, Japan, and Korea. The gonads of sea urchins are edible and contain high concentrations of protein ranging from 43.1%–45.8% (dry basis) [1]. Some studies of sea urchin *S. nudus* gonad proteins have been conducted in the field of food science. The antioxidant mechanisms and the structure–activity relationship of *S. nudus* gonad hydrolysate have been illustrated. It has been reported that *S. nudus* gonad hydrolysates prepared using papain, pepsin, neutral protease, and trypsin exhibited a reducing capacity and radical-scavenging activity, and inhibited lipid peroxidation [1,2] and paraquat-induced oxidative stress in *Caenorhabditis elegans* [3]. Furthermore, an in silico approach was used to detect peptides in the major yolk protein (MYP) of the *S. nudus* gonad, and the MYP was shown to be a good source of tryptophan-containing peptides with antioxidant activity [4].

The growth and composition of sea urchin gonads, as reflected in the gonad index (GI), show seasonal variation at the levels of free amino acid and fatty acid. Different sea urchin species attain high GI values in different months. High GI values of the sea urchins *Paracentrotus lividus* (*P. lividus*), *Evechinus chloroticus* and *Strongylocentrotus droebachiensis* (*S. droebachiensis*) were obtained from January to April [5,6], February (austral summer) [7] and between July and September [8], respectively. Seasonal variation in the amino acid constituents of sea urchin gonads has been observed in *Tripneustes gratilla* and *Diadema setosum* [9,10]. Furthermore, lipids, as one of the main components in sea urchin gonads, shows seasonal variability [11,12]. In the sea urchin *P. lividus* and *Arbacia lixula*, the gonad fatty acid profile exhibits seasonal changes [13]. An annual cyclical trend in the total lipid content of *P. lividus* gonads has also been confirmed [14]. The fatty acid composition of the *S. nudus* gonad was illustrated by Zhou et al. [15]. However, seasonal variation in the gonad protein profile of *S. nudus* has not yet been reported. Therefore, to elucidate the role of protein molecules in the changes of the sea urchin gonad condition factor, it is necessary to investigate the relationship between the GI and protein variation during the fishing season.

Proteomics techniques offer an effective approach for analyzing protein expression at the cellular level and illustrating the biological processes of organisms. In recent years, seasonal variation in the components of foodstuffs has been studied. Two-dimensional gel electrophoresis (2D), coupled with liquid chromatography tandem mass spectrometry (LC-MS/MS), is a useful technique in protein identification and quantification. In comparison with *Pueraria mirifica* tubers collected in winter, the levels of isoflavonoid biosynthesis-related proteins were higher in tubers collected in summer [16]. Moreover, the level of the protein that regulates the expression of vanadium-dependent bromoperoxidase (vBPO), and thereby helps defend the organism against bacteria and enhances its survival in summer, was shown to be upregulated in summer-collected samples of the Japanese kelp *Saccharina japonica* compared to other seasons [17]. Isobaric tagging for relative and absolute quantitation (iTRAQ), a powerful method with high throughput, was applied to seafood protein qualification analysis, coupled with LC-MS/MS. Wang et al. [18] illustrated the biochemical mechanism of razor clams (*Sinonovacula constricta*) during controlled freezing-point storage. According to Shi et al. [19], the specification of proteome changes and mechanisms provides helpful information for the property control of frozen mud shrimp. Therefore, investigation of the seasonal variation in the *S. nudus* gonad protein profile using proteomic technology is feasible.

In this study, the protein that made a difference intensity of the band between about 37 kDa in JUL–JUN group was detected by nanoLC-ESI-MS/MS. The differentially expressed proteins (DEPs) that lineally correlate with GI and protein content were identified to illustrate seasonal variations of *S. nudus* gonads during the fishing season by an iTRAQ in conjunction with an LC-MS/MS strategy. In-depth and systematic studies of the variation of the protein composition in *S. nudus* will help indicate dynamic changes in gonad condition factor and seasonal variation during the fishing season.

## 2. Results

### 2.1. GI and Protein Level of Sea Urchin Gonads

**Figure 1:**
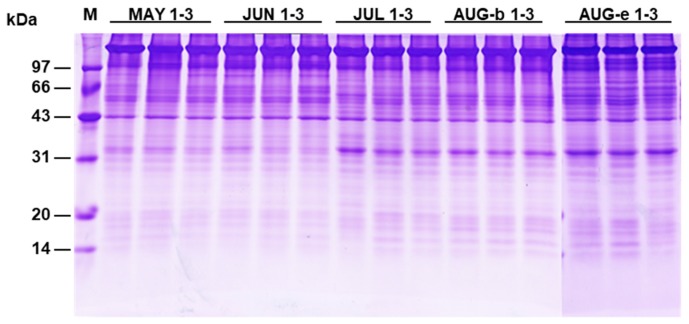
Sodium dodecyl sulfate polyacrylamide gel electrophoresis (SDS-PAGE) of gonad proteins in *S. nudus*. Lane 1, protein marker. The gonads collected in May (MAY), June (JUN), July (JUL), at the beginning of August. and at the end of August are shown in lanes 2–4, 5–7, 8–10, 11–13 and 14–16, respectively. Aug-b, the beginning of August; Aug-e, the end of August.

The GI values of the sea urchin *S. nudus* are presented in Table 1. The GI values showed no significant differences in May and June but increased gradually between July and August during the fishing season. A notable increment in the GI value was observed in the JUL group; the JUL value was 1.37-fold that of the JUN group. The AUG-e group displayed the highest GI value of 27.42% during the fishing season. The protein content did not change greatly and ranged from 11.84% to 14.02%. The AUG-e group had higher protein content than the other groups. The protein pattern of *S. nudus* gonads is shown in Figure 1. Five *S. nudus* gonad groups (MAY, JUN, JUL, AUG-b and AUG-e), with three biological replicates, showed protein bands of differing intensities at about 37 kDa. Furthermore, this protein was identified as a transforming growth factor-beta-induced protein ig-h3 (TGFBI) by nanoLC-ESI-MS/MS (data not shown).

### 2.2. Identification and Functional Classification of the DEPs

**Figure 2:**
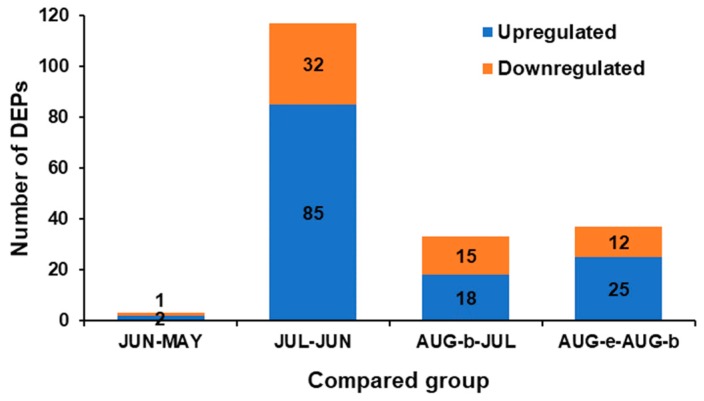
The number of upregulated and downregulated differentially expressed proteins (DEPs) identified in the compared groups (JUN–MAY, JUL–JUN, AUG-b-JUL, and AUG-e-AUG-b).

In the present study, proteomic analysis was conducted using the iTRAQ labeling method. In this way, a total of 5669 proteins in *S. nudus* gonads were identified. DEP selection was explored by the method of Li et al. [20] with some modifications. Based on the criteria of fold change (FC) (>1.2 or <0.83) and Q value (< 0.05) and Protein-level FDR <= 0.01, a total of 174 DEPs were identified in the compared groups (Figure 2). The numbers of DEPs in the JUN–MAY, JUL–JUN, AUG-b-JUL, and AUG-e-AUG-b groups were 3, 117, 33, and 37, respectively. The JUN–MAY group contained the fewest DEPs (2 upregulated proteins and 1 downregulated protein), indicating that the fewest changes in *S. nudus* proteins were observed in May and June. In comparison with the JUN group, the JUL group possessed 117 DEPs, indicating that the greatest change in the number of regulated proteins occurred between June and July, which was followed by the AUG-e-AUG-b group. Therefore, further investigation into the functional classification of these two groups was needed.

**Figure 3:**
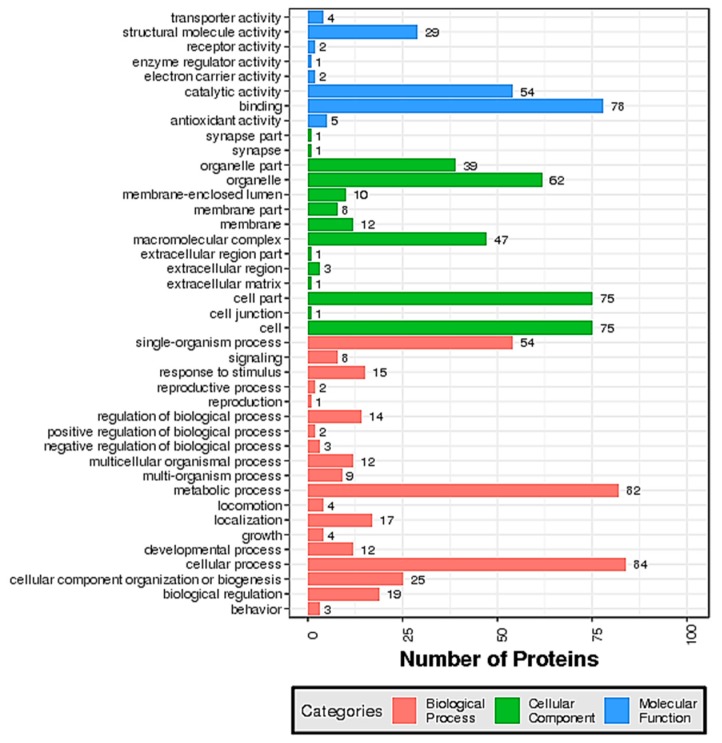
Gene ontology (GO) annotation of the upregulated and downregulated differentially expressed proteins (DEPs) identified in compared groups (JUN–MAY, JUL–JUN, AUG-b-JUL, and AUG-e-AUG-b) including biological processes, molecular functions, and cellular components.

Bioinformatics analysis is an extensive method that can be used to understand the classification of proteins. The Gene Ontology (GO) database consists of three ontologies, including biological process, cellular component, and molecular function. The GO distribution of the 174 filtered DEPs is shown in Figure 3. In the category of biological processes, the single-organism process, metabolic process, and cellular process were the main three annotated biological processes associated with the DEPs. The DEPs in the category of cellular components were involved in organelles, the macromolecular complex, and cells. In the molecular function group, the proteins mainly possessed structural molecule activity, catalytic activity and binding activity.

The functional classification of the JUL–JUN and AUG-e-AUG-b groups was investigated. We also observed significant regulation of the GI values in these two groups (Table 1). In the JUL–JUN and AUG e-AUG-b groups, the GO term showed significant enrichment in 117 and 37 kinds of DEPs, respectively (Figure 2). The GO term with more than 30% of DEPs in the foreground is summarized in Table 2 and Table 3. In the JUL–JUN group, the DEPs in the category of biological process were involved in the metabolic process, cellular metabolic process, and organic substance metabolic process. The DEPs in the category of cellular components were mainly located in cytoplasm, intracellular organelles, and organelles. According to the GO annotation, the heterocyclic compound binding, organic cyclic compound binding, structural molecule activity, and structural constituency of the ribosomes were located in the molecular function group. In the AUG-e-AUG-b group, biological process analysis revealed that the DEPs were involved in the cellular nitrogen compound’s metabolic process, the nucleobase-containing compound metabolic process, and the biosynthetic process. Cellular component analysis indicated that the DEPs were associated with cytosol and cytosolic parts. As for the molecular function ontology, the DEPs were located in the heterocyclic compound binding, organic cyclic compound binding, and small molecule binding.

### 2.3. Correlations between Differentially Expressed Proteins (DEPs) and the Gonad Index (GI) and Protein Content

Among the 174 DEPs, TGFBI with a high expression level from July, as well as the other 14 DEPs that appeared at least twice, were chosen for further analysis. The relationship between the 14 filtered DEPs and the GI and protein content of *S. nudus* gonads is shown in Table 4. Pearson’s correlation analysis was performed to further screen the DEPs. Of the 15 DEPs, only 10 were significantly correlated (*P* < 0.05 or 0.01) with protein content, and 7 were significantly correlated with both GI and protein content. However, 5 of the DEPs showed no correlation with either GI or protein content. Interestingly, all 10 DEPs were concentrated in both JUL–JUN and AUG-e-AUG-b groups. Among them, the GO terms of these 10 DEPs were all reflected in Table 2 and Table 3. The DEPs that showed a correlation with GI or protein content might be involved in regulating the *S. nudus* gonads’ condition factor during the fishing season. Although the FC of TGFBI increased to 2.17 in the JUL–JUN group, the protein didn not show any correlation with the GI or protein content (Table 4). Therefore, it is necessary to illustrate in detail the characteristics of the 10 DEPs in *S. nudus* gonads during the fishing season.

### 2.4. Potential Protein Markers Associated with the GI and Protein Content of S. nudus Gonads

#### 2.4.1. Metabolic Enzyme

6-phosphogluconate dehydrogenase (6PGD) is an oxidative carboxylase that is found in many cells and tissues. 6PGD showed significantly positive correlations with GI (*P* < 0.05) and protein content (*P* < 0.01). The upregulation of 6PGD was observed in the JUL–JUN and AUG-e-AUG-b groups with an FC of 1.24 and 1.27, respectively (Table 4). 

Isocitrate dehydrogenase (IDH) exists in mitochondria and catalyzes the oxidative decarboxylation of isocitrate. In the present study, IDH expression correlated positively with both GI and protein content (*P* < 0.01). The upregulation of the IDH was observed in the JUL–JUN and AUG-e-AUG-b groups with FCs of 1.29 and 1.20, respectively (Table 4).

Aldehyde dehydrogenase (ALDH) is an NAD-dependent oxidoreductase that converts aldehydes to carboxylic acids by oxidation. In this study, ALDH expression showed a negative correlation with both the GI and protein content (*P* < 0.01). An FC of 0.82 was observed in the IDH downregulated JUL–JUN and AUG-e-AUG-b groups (Table 4). 

Choline dehydrogenase (CHDH) is an enzyme that catalyzes the conversion of choline to betaine aldehyde. CHDH showed a negative correlation with protein content (*P* < 0.01) but was not significantly correlated with the GI. The downregulation of the CHDH was observed in the JUL–JUN and AUG-e-AUG-b groups with FCs of 0.81 and 0.82, respectively (Table 4).

#### 2.4.2. Biosynthesis Enzyme

CAD protein (carbamoyl-phosphate synthetase 2, aspartate transcarbamylase, and dihydroorotase) is an enzyme that is involved in pyrimidine biosynthesis. In our study, CAD protein expression was positively correlated with the GI and protein content (*P* < 0.05). The upregulation of the CAD protein was observed in the JUL–JUN and AUG-e-AUG-b groups with FCs of 1.21 and 1.31, respectively (Table 4).

The multifunctional protein ADE2, is an important enzyme that participates in nucleotide synthesis. Multifunctional protein ADE2 isoform X3 showed positive correlations with both the GI and protein content (*P* < 0.01). The upregulation of the multifunctional protein ADE2 isoform X3 was observed in the JUL–JUN and AUG-e-AUG-b groups with FCs of 1.33 and 1.28, respectively (Table 4).

Ribonucleoside-diphosphate reductase (RRM1) is an essential enzyme that produces deoxyribonucleotides. In this study, RRM1 expression showed a positive correlation with protein content (*P* < 0.01) but had no correlation with the GI. The upregulation of the RRM1 was observed in the JUL–JUN and AUG-e-AUG-b groups with FCs of 1.32 and 1.40, respectively (Table 4).

#### 2.4.3. Structural Protein

Myoferlin is a muscle-specific protein belonging to the Ferlin family and is essential for endocytosis by endothelial cells. Myoferlin isoform X8, an isoform of myoferlin, exhibited a positive correlation with the GI and protein content (*P* < 0.01). The upregulation of the myoferlin was observed in the JUL–JUN and AUG-e-AUG-b groups with FCs of 1.43 and 1.31, respectively (Table 4).

#### 2.4.4. Ribosomal Protein

Ribosomal protein is a protein that, in conjunction with rRNA, forms the ribosomal subunits that are necessary for the cellular process of translation. The results of this study revealed that ribosomal protein L36 (RL36) exhibited a significant positive correlation with protein content (*P* < 0.05). An FC of 1.24 was observed in the ribosomal protein upregulated JUL–JUN and AUG-e-AUG-b groups (Table 4).

#### 2.4.5. Functional Peptides

Sperm-activating peptides (SAPs) secreted by sea urchin eggs are diffusible in seawater, and sea urchin sperm are activated by SAPs, which promote the activation of the sperm and attract the sperm to the eggs [21]. SAP expression showed a significant positive correlation with both the GI and protein content (*P* < 0.01). The upregulation of SAPs was observed in the JUL–JUN and AUG-e-AUG-b groups with FCs of 2.03 and 1.49, respectively (Table 4).

### 2.5. Protein–Protein Interaction (PPI) Analysis

**Figure 4:**
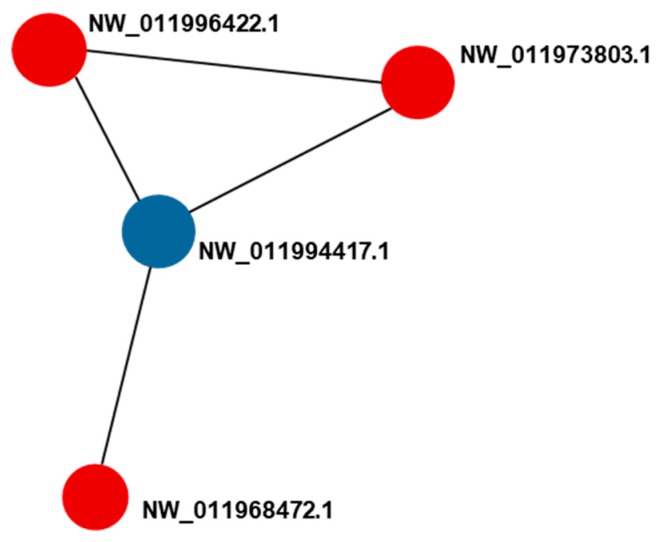
The protein-protein interaction (PPI) analysis among the differentially expressed proteins (DEPs) related to both gonad index (GI) and protein content in the compared groups (JUN–MAY, JUL–JUN, AUG-b-JUL, and AUG-e-AUG-b). Red nodes represent upregulated proteins; blue nodes represent downregulated proteins; black line: co-expression.

PPIs are interactions between more than one protein molecule, which participate in biological processes and are influenced by the hydrophobic effect. PPI-mediated events occur in the tissues and cells of living organisms under various biomolecular circumstances. String 11.0 was used to estimate the relationships between the 7 DEPs that showed significant correlations with both GI and protein content. Figure 4 shows the protein names and the interactions among these DEPs. The red and blue nodes indicate DEPs in the *S. nudus* gonad that show up- and downregulated expression, respectively, and the connecting lines indicate the type of reaction. Four of the screened DEPs, including 6PGD, IDH, ALDH, and the multifunctional protein ADE2, had connections with each other.

## 3. Discussion

The GI index of sea urchins is a good indicator of mature status and reproductive cycle. Moreover, the development of nutritive phagocytes in sea urchin gonads reflected reproductive cycle phases, which could trigger changes in the GI level [22]. With increases of the *S. nudus* GI, the condition factor of sea urchin gonads also increased in the storage period, and the maturity of the gametogenesis was also enhanced. Protein is not only a key role in providing energy but also an important substance basis for development. Sea urchin gonad protein content can vary during the period of gametogenesis [23,24,25]. According to Archana et al. [26], sea urchin gonads are a good source of protein, which can provide energy and essential amino acids.

The seasonal cycle of the GI of *S. nudus* has been reported previously. Nevertheless, timing differences were observed between the GI peaks in both the literature and the present study in the range of May–August [27]. These peaks may be due to different sea urchin provenances. A different proportion of GI increasement was observed for both *S. nudus* and other sea urchin species [28,29]. Feed type, feed intake, and water temperature are the main factors that have an impact on sea urchin GI variation [30]. Similar to our results, the GI values of *Pseudocentrotus depressus* were enhanced during the fishing season [31]. Moreover, the gonad condition factor of the organism could be indicated by its GI, and the protein content contributed to the GI increment [32]. The observed variation in GI and protein content illustrates that *S. nudus* gonad maturity is a dynamic process [33]. 

In the present study, GI and protein content were found to correlate with different proteins, including metabolic enzymes, biosynthesis enzymes, structural proteins, ribosomal proteins, and functional proteins. Thus, the DEPs that have a correlation with the GI or protein content are illustrated.

6PGD forms pentose phosphate from hexose phosphate, providing energy for cellular functions [34]. In sea urchin eggs, 6PGD catalyzes the decarboxylating reduction of 6-phosphogluconate [35]. In our study, the overabundance of 6PGD might be an adaptation that increases energy metabolism in *S. nudus* gonad development during the fishing season. IDH participates in NADP^+^ circulation in the mitochondria [36]. Moreover, the level of IDH in *Strongylocentrotus purpuratus* eggs was also affected by solar ultraviolet radiation and glutathionylation during periods of oxidative stress [37,38]. Therefore, IDH in *S. nudus* gonads is an enzyme that participates in redox reactions and may be susceptible to ultraviolet radiation during the fishing season. It has been reported that ALDH can counteract the toxicity caused by ethanol metabolism or lipid peroxidation in sea bream liver, and the activity of ALDH in rat ovaries was shown to be lower than that in liver [39,40]. Hence, ALDH in *S. nudus* gonads was deduced to play a role in the oxidization of aldehydes to carboxylic acids during the fishing season. CHDH is found in rat reproductive cells and has been shown to participate in mouse oocyte meiotic maturation. The deletion of CHDH decreased rat sperm motility [41,42]. Thus, the CHDH found in *S. nudus* gonads was deduced to take part in reproductive cell development during the fishing season.

CAD protein not only particiaptes in synthesising pyrimidine, but also regulates Notch/Vegf signaling and vascular development in zebrafish [43]. Therefore, it can be deduced that the CAD protein in *S. nudus* gonads may participate in pyrimidine biosynthesis and biological information expression during the fishing season. Multifunctional protein ADE2, which is found in hapuku eggs and in the nematocyst of the jellyfish *Stomolophus meleagris*, has been reported to be involved in nucleotide synthesis [44,45]. In the present study, the upregulation of multifunctional protein ADE2 isoform X3 in *S. nudus* gonads may reflect the elevated protein content of the gonads, owing to the increment of nuclear transport during the fishing season. RRM1 is an essential enzyme that produces deoxyribonucleotides that are used in DNA synthesis in cells. Moreover, it is regarded as a biomarker in lung cancer [46]. It is speculated that the content of deoxyribonucleotides decreased during *S. nudus* development. Therefore, RRM1 was deduced to participate in generating deoxyribonucleotides in *S. nudus* gonads during the fishing season. Myoferlin was also detected in the plasma membrane of mature oocytes of sea stars (*Patiria miniate*), an echinoderm [47]. In the present study, the upregulation of myoferlin isoform X8 was inferred to facilitate endocytosis via *S. nudus* gonads endothelial cells during the fishing season.

In previous studies, the expression of RL36 has been shown to correlate with temperature-sensitive growth in *Saccharomyces cerevisiae* [48]. RL36 was also detected during the development of the Pacific oyster *Crassostrea gigas* [49]. Thus, RL36 in *S. nudus* gonads may participate in ribosomal subunit formation and may be affected by the temperature during individual developments during the fishing season. Sperm activated by SAPs was observed to have higher intracellular pH and Ca^2+^ concentrations [50]. It is speculated that the increased content of SAPs could be related to the condition factor of *S. nudus* gonads during the fishing season.

The results indicated that 6PGD, IDH, ALDH, and the multifunctional protein ADE2 might participate in the changes of *S. nudus* gonads’ condition factor together during the fishing season. In the previous study, 6PGD and IDH were demonstrated to be coregulated in rat livers and hearts [51]. 6PGD and ALDH were also shown to be coregulated in *Citrus sinensis* roots [52]. Regulation of IDH and ALDH was observed in human pathological groups [53]. Furthermore, both ALDH and multifunctional protein ADE2 were regulated in control and treated leukemic cells [54]. 

## 4. Materials and Methods

### 4.1. Sea Urchin Collection and Gonad Preparation

Fresh 3 year-old sea urchins (*S. nudus*) (body weight, 172.24–195.55 g, test diameter, 7.65–7.81 cm) were purchased every three weeks from May. Then, sea urchins were collected in June, July, and at the beginning and end of August, 2018, from a local market in Dalian, China. The age of sea urchins can be calculated by counting growth rings on the rotula of the Aristotle’s lantern complex [55]. The cleaned rotula was broken into two parts along the short axis. Growth rings were observed in a cross section after the two parts of rotula were charred. One light and one dark band was considered to be one pair of rings, which was considered to represent one year.

In the present research, the protocol was approved by the Animal Care and Ethics Committee of Dalian Medical University. All animal experiment protocols followed accepted standards of humane animal care and were in line with the guidelines of the U.S. National Institutes of Health Guide for the Care and Use of Laboratory Animals as well as the recommendations established by the Animal Care and Use Committee of Dalian Medical University.

Twenty sea urchins were immediately transferred to the laboratory for analysis. The sea urchins were weighed and dissected, and the gonads were then entirely removed and weighted. A GI was calculated for each individual as follows:

GI (%) = wet gonad mass/wet body mass × 100

The average GI of 20 *S. nudus* was calculated in each sampling. Then, 12 individuals, which were closer to the average value, were screened out for further analysis.

Sea urchin gonads was washed with phosphate-buffered saline (0.137 mM NaCl, 2.7 mM KCl, 10 mM Na_2_HPO_4_ and 2 mM KH_2_PO_4_) and placed in 2 mL cryogenic vials. The vials were immediately frozen in liquid nitrogen and stored at −80 ℃ until analysis. The Kjeldahl method was used to determine the protein content (conversion factor, %N × 6.25) according to the method of Agatsuma et al. [56].

### 4.2. Extraction of Sea Urchin Gonad Protein

**Figure 5:**
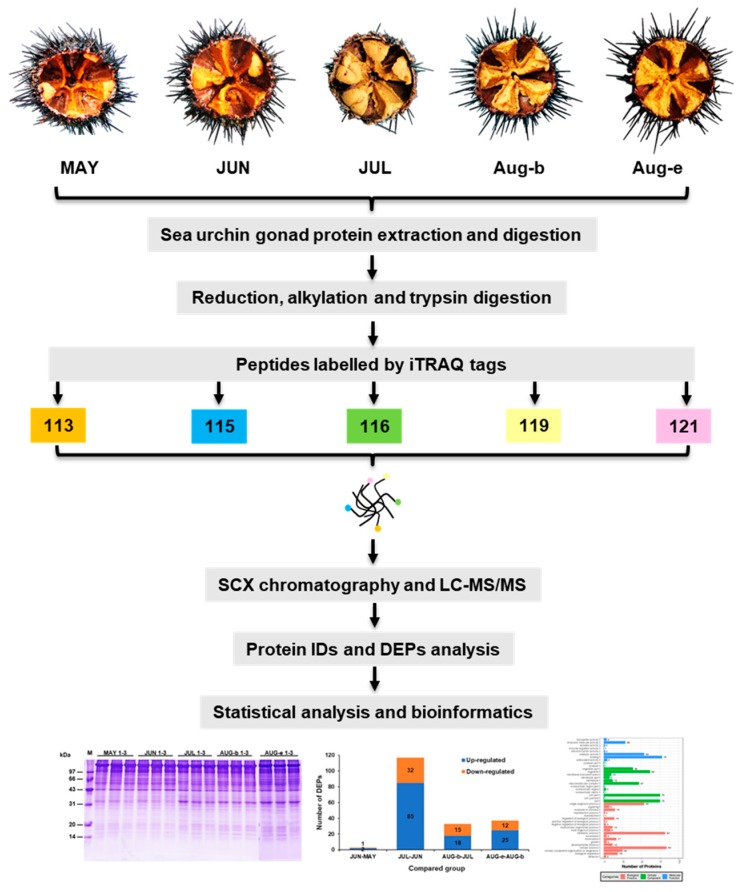
Experimental design and workflow for the quantitative proteomic analysis of *S. nudus* gonads using the isobaric tagging for relative and absolute quantitation (iTRAQ) labelling proteomic strategy.

To avoid the differences among the individuals, sample pooling method was used and create accurate data in proteomic studies [57]. Therefore, an aliquot of gonads from 4 sea urchins were pooled in each replicate and each group consisted of three biological replicates. In each replicate, an *S. nudus* gonad sample with the same weight was taken from each sea urchin. Then, the gonads were mixed together and homogenized for further analysis. The workflow of the research is illustrated in Figure 5.

Twenty-five milligrams of *S. nudus* gonads were mixed with 1 mL lysis buffer 3 (8 M urea, 40 mM tetraethyl ammonium bromide (TEAB), pH 8.5), containing 1 mM phenylmethanesulfonyl fluoride (PMAF) and 2 mM ethylenediaminetetraacetic acid (EDTA) solution (final concentration). After cooling in ice for 5 min, 10 mM DTT solution was added to the solution (final concentration) followed by 1 min sonication (200 W) and centrifugation (25,000 g, 4 °C, 20 min). After incubation (56 °C, 1 h), 55 mM iodoacetamide (IAM) (final concentration) was added to the sediment. The alkylation reaction was allowed to proceed in the dark (45 min), and the sediment was then placed in 5 volumes of chilled acetone. After storage at −20 °C for 2 h, the precipitate was collected by centrifugation (25,000 g, 4 °C, 20 min). This procedure was repeated until the supernatant became clear. The residue was dissolved in a lysis buffer 3 with sonication (200 W) on ice for 1 min; the samples were then centrifuged (25,000 g, 4 °C, 20 min), and the protein concentration of the supernatant was determined using a Bovine Serum Albumin (BSA) kit.

### 4.3. SDS-PAGE

The protein profile of the extracted proteins was obtained by SDS-PAGE, according to the protocol of Wu et al. [58] with some modifications. Briefly, 20 μL of each protein sample with loading buffer added was heated at 95 °C for 5 min and then centrifuged at 25,000 g for 5 min. The supernatant was loaded on a 12% separating gel, which was then run at 120 V for 120 min. After staining with coomassie brilliant blue solution R-250 for 2 h, the gel was placed in a destaining solution consisting of ethanol and acetic acid (4: 1, v/v) until the protein bands were clearly visible.

### 4.4. Trypsin Digestion and iTRAQ Labeling

Trypsin digestion was conducted according to Cao et al. [59], with some modifications. A sample containing 100 μg of sea urchin gonad protein in 8 M urea was diluted with 100 mM TEAB. The solution was mixed with 2.5 μg trypsin (Promega, Madison, WI, USA) at a ratio of 40: 1 (v/v) and incubated at 37 ℃ for 4 h. After incubation, the solution was desalted by passage over a Strata X column, and the eluted peptide solution was freeze-dried. The obtained peptides were dissolved in 30 μL 0.5 M TEAB and labeled using iTRAQ Reagent 8 plex kits (AB Sciex, Redwood City, CA, USA), according to the manufacturer’s protocol. After incubation for 2 h at room temperature, a Strata X C18 column (Phenomenex) was used to desalt the samples. The samples were vacuum-dried according to the method recommended by the manufacturer.

### 4.5. Peptide Fractionation by Strong Cation Exchange (SCX) Chromatography Separation

Peptide fractionation by strong cation exchange (SCX) chromatography separation was conducted according to Cao et al. [59]. The collected peptides were dissolved in 2 mL buffer A (5% acetonitrile (ACN), 95% H_2_O, adjusted to pH 9.8 with ammonia), and the solution was loaded onto a column containing 5 μm particles (Phenomenex). The peptides were separated by gradient elution in buffer B (5% H_2_O, 95% ACN, adjusted to pH 9.8 with ammonia) as follows: 5% buffer B for 10 min, 5%–35% buffer B for 40 min, and 35%–95% buffer B for 1 min. The flow rate was 1 mL/min. Buffer B at a concentration of 95% flowed through the HPLC system for 3 min, and the concentration of buffer B was then reduced to 5% over a period of 1 min. The system was equilibrated with 5% buffer B for the next 10 min, and the elution was monitored at 214 nm. The eluted peptides were separated into 20 fractions and vacuum dried.

### 4.6. High Pressure Liquid Chromatography (HPLC) Coupled with Mass Spectrometer (MS) Detection

HPLC-MS detection was conducted by the method of Cao et al. [59]. Each peptide sample was resuspended in buffer A (2% ACN, 0.1% formic acid), and the supernatant was collected after centrifugation of the sample at 20,000 g for 10 min. The solution was eluted at 5 μL/min for 8 min in a Thermo Scientific™ UltiMate™ 3000 UHPLC system (Thermo Fisher Scientific, San Jose, CA, USA). The sample was then loaded onto a nanocapillary C18 column (ID 75 μm × 25 cm, 3 μm particles) at a flow rate of 300 nL/min. Buffer B (98% ACN, 0.1% FA) was used for a gradient elution. Buffer B was increased from 5% to 25% over a period of 40 min and then increased to 35% over the next 5 min. Then, buffer B was increased to 80% over a period of 2 min and maintained for 2 min. Finally, buffer B was returned to 5% over a period of 1 min and maintained for 6 min.

The eluted fraction was subjected to tandem mass spectrometry Q EXACTIVE HF X coupled with nanoelectrospray ionization (Thermo Fisher Scientific, San Jose, CA, USA) for DDA (data-dependent acquisition) detection. MS spectral analysis was conducted using the following parameters: electrospray voltage: 2.0 kV; precursor scan range: 350–1500 m/z at a resolution of 60,000 in Orbitrap; MS/MS fragment scan range: >100 m/z at a resolution of 15,000 in HCD mode; normalized collision energy setting: 30%; dynamic exclusion time: 30 s; automatic gain control (AGC) for full MS.

### 4.7. Database Search and iTRAQ Quantification Analysis

Data analysis was performed using the Thermo Scientific tool Proteome Discoverer coupled with Mascot version 2.3.02 software against the National Center for Biotechnology Information (NCBI) database (last accessed on November 2018) from the NCBI website (https://www.ncbi.nlm.nih.gov/). At least one unique peptide was necessary for the identified protein. According to Wen et al. [60], IQuant, which integrates the Mascot Percolator, was used to quantitatively analyze isobaric tag-labeled peptides. A PSM-level false discovery rate (FDR) of 1% was used to filter the peptides of confidence. The identified peptide sequences were assembled into proteins with confidence using the “simple principle” strategy. The false-positive rate at the protein level was controlled at an FDR of 1% (Protein-level FDR <= 0.01) [61].

### 4.8. Bioinformatic and Statistical Analysis

The significantly regulated DEPs were selected based on a ratio of FC > 1.2 (or <0.83) and a student’s -t test *P* (< 0.05) value [34]. GO enrichment (http://www.geneontology.org/) was employed to filter the DEPs. Pearson’s correlations analysis between DEPs and the GI and protein content of *S. nudus* gonads was conducted using SPSS 19.0. PPI analysis was conducted using the String database. Each experiment was performed three times. The results are shown as the means ± standard deviation (SD). Bonferroni correction was used in the statistical analysis.

## 5. Conclusions

Proteomic analysis was applied to detect seasonal variation in protein expression in *S. nudus* gonads during the fishing season. The mechanism of *S. nudus* gonads that changes the condition factor was revealed by screening, as well as the characteristics of the 10 DEPs that had a correlation with GI and protein content. This study offers valuable evidence that will lead to a better understanding of the mechanisms of the *S. nudus* gonad condition factor during the fishing season.

## Figures and Tables

**Table 1:** Gonad index (GI) and protein content of sea urchin *Strongylocentrotus nudus*.

-	MAY	JUN	JUL	AUG-b	AUG-e
Gonad index (%)	16.56 ± 2.63^c^	15.86 ± 2.35^c^	21.80 ± 2.19^b^	22.80 ± 1.31^b^	27.42 ± 2.31^a^
Protein content (%)	12.32 ± 0.23^bc^	11.84 ± 0.33^c^	13.64 ± 0.78^ab^	13.03 ± 0.67^abc^	14.20 ± 0.44^a^

Data represent the mean ± one standard deviation (n = 12). The different letters in the same line indicate a significant difference at *P* < 0.004.

**Table 2:** Description of Gene Ontology (GO) terms of the differentially expressed proteins (DEPs) in the JUL–JUN group.

Gene Ontology Term	M	m	Percent in Foreground	N	n	Percent in Foreground	E-ratio	P-value
**Biological process**								
metabolic process (GO:0008152)	62	47	75.81	2428	1428	58.81	30.38	0.0035
cellular metabolic process (GO:0044237)	62	42	67.74	2428	1207	49.71	28.74	0.0028
organic substance metabolic process (GO:0071704)	62	42	67.74	2428	1266	52.14	30.14	0.0085
primary metabolic process (GO:0044238)	62	41	66.13	2428	1171	48.23	28.56	0.0031
nitrogen compound metabolic process (GO:0006807)	62	40	64.52	2428	1065	43.86	26.63	0.0007
organonitrogen compound metabolic process (GO: 1901564)	62	35	56.45	2428	743	30.6	21.23	1.67 × 10^−5^
cellular nitrogen compound metabolic process (GO: 0034641)	62	34	54.84	2428	699	28.79	20.56	1.20 × 10^−5^
cellular macromolecule metabolic process (GO:0044260)	62	30	48.39	2428	749	30.85	24.97	0.0025
macromolecule metabolic process (GO:0043170)	62	30	48.39	2428	802	33.03	26.73	0.0079
cellular biosynthetic process (GO:0044249)	62	29	46.77	2428	499	20.55	17.21	2.42 × 10^−6^
organic substance biosynthetic process (GO:1901576)	62	29	46.77	2428	502	20.68	17.31	2.76 × 10^−6^
biosynthetic process (GO:0009058)	62	29	46.77	2428	537	22.12	18.52	1.16 × 10^−5^
cellular nitrogen compound biosynthetic process (GO:0044271)	62	27	43.55	2428	322	13.26	11.93	2.56 × 10^−9^
organonitrogen compound biosynthetic process (GO:1901566)	62	27	43.55	2428	340	14	12.59	8.88 × 10^−9^
cellular protein metabolic process (GO:0044267)	62	27	43.55	2428	445	18.33	16.48	2.92 × 10^−6^
protein metabolic process (GO:0019538)	62	27	43.55	2428	483	19.89	17.89	1.50 × 10^−5^
cellular macromolecule biosynthetic process (GO:0034645)	62	25	40.32	2428	296	12.19	11.84	1.17 × 10^−8^
macromolecule biosynthetic process (GO:0009059)	62	25	40.32	2428	297	12.23	11.88	1.26 × 10^−8^
gene expression (GO:0010467)	62	25	40.32	2428	394	16.23	15.76	3.71 × 10^−6^
translation (GO:0006412)	62	24	38.71	2428	146	6.01	6.08	1.09 × 10^−14^
peptide biosynthetic process (GO:0043043)	62	24	38.71	2428	150	6.18	6.25	2.07 × 10^-14^
amide biosynthetic process (GO:0043604)	62	24	38.71	2428	158	6.51	6.58	7.01 × 10^−14^
peptide metabolic process (GO:0006518)	62	24	38.71	2428	175	7.21	7.29	7.47 × 10^−13^
cellular amide metabolic process (GO:0043603)	62	24	38.71	2428	203	8.36	8.46	2.11 × 10^−11^
**Cellular component**								
cytoplasm (GO:0005737)	54	40	74.07	2540	1309	51.54	1.44	0.0005
intracellular organelle (GO:0043229)	54	40	74.07	2540	1385	54.53	1.36	0.0023
organelle (GO:0043226)	54	40	74.07	2540	1456	57.32	1.29	0.0077
cytoplasmic part (GO:0044444)	54	36	66.67	2540	945	37.2	1.79	8.71 × 10^−6^
non-membrane-bounded organelle (GO:0043228)	54	33	61.11	2540	475	18.7	3.27	3.71 × 10^−12^
intracellular non-membrane-bounded organelle (GO:0043232)	54	33	61.11	2540	475	18.7	3.27	3.71 × 10^−12^
macromolecular complex (GO:0032991)	54	30	55.56	2540	842	33.15	1.68	0.0005
intracellular ribonucleoprotein complex (GO:0030529)	54	29	53.7	2540	246	9.69	5.55	1.57 × 10^−16^
ribonucleoprotein complex (GO:1990904)	54	29	53.7	2540	246	9.69	5.55	1.57 × 10^−16^
ribosome (GO:0005840)	54	27	50	2540	104	4.09	12.21	8.25 × 10^−25^
intracellular organelle part (GO:0044446)	54	26	48.15	2540	884	34.8	1.38	0.0282
organelle part (GO:0044422)	54	26	48.15	2540	894	35.2	1.37	0.0325
ribosomal subunit (GO:0044391)	54	19	35.19	2540	76	2.99	11.76	8.65 × 10^−17^
cytosol (GO:0005829)	54	19	35.19	2540	268	10.55	3.33	8.96 × 10^−7^
cytosolic ribosome (GO:0022626)	54	17	31.48	2540	57	2.24	14.03	1.85 × 10^−16^
cytosolic part (GO:0044445)	54	17	31.48	2540	80	3.15	10	9.69 × 10^−14^
**Molecular function**								
heterocyclic compound binding (GO:1901363)	72	35	48.61	3011	1154	38.33	1.27	0.0464
organic cyclic compound binding (GO:0097159)	72	35	48.61	3011	1155	38.36	1.27	0.0469
structural molecule activity (GO:0005198)	72	26	36.11	3011	152	5.05	7.15	8.91 × 10^−17^
structural constituent of ribosome (GO:0003735)	72	24	33.33	3011	90	2.99	11.15	2.74 × 10^−20^

N: number of proteins in the background annotated by at least one GO term; n: number of proteins in the background annotated by the GO term; M: number of DEPs annotated by at least one GO term; m: number of DEPs annotated by the GO term; E-ratio: the enrichment ratio; *P*-value: the GO term shows significant enrichment in DEPs (*P* < 0.05).

**Table 3:** Description of the Gene Ontology (GO) terms of the differentially expressed proteins (DEPs) in the AUG e–AUG-b group.

Gene Ontology Term	M	m	Percent in Foreground	N	n	Percent in Foreground	E-ratio	P-value
**Biological process**								
cellular nitrogen compound metabolic process (GO:0034641)	18	10	55.56	2428	699	28.79	1.93	0.0153
nucleobase-containing compound metabolic process (GO:0006139)	18	8	44.44	2428	522	21.5	2.07	0.0243
biosynthetic process (GO:0009058)	18	8	44.44	2428	537	22.12	2.01	0.0286
cellular aromatic compound metabolic process (GO:0006725)	18	8	44.44	2428	554	22.82	1.95	0.0341
heterocycle metabolic process (GO:0046483)	18	8	44.44	2428	554	22.82	1.95	0.0341
organic cyclic compound metabolic process (GO:1901360)	18	8	44.44	2428	575	23.68	1.88	0.042
**Cellular component**								
cytosol (GO:0005829)	13	4	30.77	2540	268	10.55	2.92	0.0402
cytosolic part (GO:0044445)	13	4	30.77	2540	80	3.15	9.77	0.0005
**Molecular function**								
heterocyclic compound binding (GO:1901363)	20	13	65	3011	1154	38.33	1.7	0.014
organic cyclic compound binding (GO:0097159)	20	13	65	3011	1155	38.36	1.69	0.0141
small molecule binding (GO:0036094)	20	11	55	3011	697	23.15	2.38	0.002
nucleotide binding (GO:0000166)	20	10	50	3011	647	21.49	2.33	0.0044
nucleoside phosphate binding (GO:1901265)	20	10	50	3011	647	21.49	2.33	0.0044
anion binding (GO:0043168)	20	9	45	3011	692	22.98	1.96	0.0241
oxidoreductase activity (GO:0016491)	20	8	40	3011	333	11.06	3.62	0.0008
ATP binding (GO:0005524)	20	7	35	3011	421	13.98	2.5	0.0149
adenyl ribonucleotide binding (GO:0032559)	20	7	35	3011	427	14.18	2.47	0.0161
adenyl nucleotide binding (GO:0030554)	20	7	35	3011	428	14.21	2.46	0.0163
drug binding (GO:0008144)	20	7	35	3011	463	15.38	2.28	0.0245

N: number of proteins in the background annotated by at least one GO term; n: number of proteins in the background annotated by the GO term; M: number of DEPs annotated by at least one GO term; m: number of DEPs annotated by the GO term; E-ratio: the enrichment ratio; *P*-value: the GO term shows significant enrichment in DEPs (*P* < 0.05).

**Table 4:** Pearson’s correlation between differentially expressed proteins (DEPs), gonad index (GI) and protein content during the fishing season of *Strongylocentrotus nudus*.

Protein ID ^1^	Gene ID^1^	Description	Upregulated Groups (Fold Change)	Downregulated Groups (Fold Change)	Correlation	
					GI	Protein Content
XP_797409.2	NW_011993034.1	transforming growth factor-beta-induced protein ig-h3	JUL–JUN (**2.17**)	-	0.387	0.640
XP_011676765.1	NW_011992289.1	uncharacterized protein LOC584238 isoform X2	JUN–MAY (**1.20**) AUG-b-JUL (**1.23**)	AUG-e-AUG-b (**0.76**)	-0.357	-0.511
XP_003727927.1	NW_011996422.1	6-phosphogluconate dehydrogenase, decarboxylating isoform X2	JUL–JUN (**1.24**) AUG-e-AUG-b (**1.27**)	-	0.952 *	0.959 **
XP_011667092.1	NW_011975415.1	CAD protein	JUL–JUN (**1.21**) AUG-e-AUG-b (**1.31**)	-	0.92 *	0.92 *
XP_011667652.1	NW_011976138.1	myoferlin isoform X8	JUL–JUN (**1.43**) AUG-e-AUG-b (**1.31**)	-	0.97 **	0.975 **
XP_795341.2	NW_011973770.1	annexin A7	JUL–JUN (**1.91**)	AUG-b-JUL (**0.63**)	0.503	0.747
NP_001229579.1	NM_001242650.1	ribosomal protein L36	JUL–JUN (**1.24**) AUG-e-AUG-b (**1.24**)	-	0.784	0.92 *
XP_011665962.1	NW_011973803.1	isocitrate dehydrogenase [NADP], mitochondrial isoform X2	JUL–JUN (**1.29**) AUG-e-AUG-b (**1.20**)	-	0.97 **	0.991 **
XP_003724739.1	NW_011968472.1	multifunctional protein ADE2 isoform X3	JUL–JUN (**1.33**) AUG-e-AUG-b (**1.28**)	-	0.979 **	0.988 **
XP_780425.1	NW_011996844.1	ribonucleoside-diphosphate reductase large subunit	JUL–JUN (**1.32**) AUG-e-AUG-b (**1.40**)	-	0.9	0.958 **
NP_999771.1	NW_011983912.1	sperm-activating peptides	JUL–JUN (**2.03**) AUG-e-AUG-b (**1.49**)	JUN–MAY (**0.76**)	0.994 **	0.937 *
XP_011678336.1	NW_011992922.1	uncharacterized protein LOC105445027	AUG-b-JUL (**1.66**)	AUG-e-AUG-b (**0.69**)	0.188	-0.025
XP_786787.2	NW_011994417.1	aldehyde dehydrogenase, mitochondrial	-	JUL–JUN (0.82) AUG-e-AUG-b (**0.82**)	-0.997 **	-0.935 *
XP_796478.2	NW_011979615.1	choline dehydrogenase, mitochondrial-like	-	JUL–JUN (**0.81**) AUG-e-AUG-b (**0.82**)	-0.848	-0.967 **
XP_011679601.1	NW_011993533.1	catalase-like, partial	-	JUL–JUN (0.79) AUG-e-AUG-b (0.80)	-0.519	-0.746

^1^ National Center for Biotechnology Information (NCBI) database: https://www.ncbi.nlm.nih.gov/; “-” means no up-regulation or down-regulation of the DEPs was observed in the compared groups; the superscript of “*” of data means a significant correlation (*P* < 0.05), while “**” means extremely significant correlation (*P* < 0.01).
